# Transcatheter Arterial Embolization Alone for Giant Hepatic Hemangioma

**DOI:** 10.1371/journal.pone.0135158

**Published:** 2015-08-19

**Authors:** Jun-Hui Sun, Chun-Hui Nie, Yue-Lin Zhang, Guan-Hui Zhou, Jing Ai, Tan-Yang Zhou, Tong-Yin Zhu, Ai-Bin Zhang, Wei-Lin Wang, Shu-Sen Zheng

**Affiliations:** 1 Hepatobiliary and Pancreatic Interventional Treatment Center, Division of Hepatobiliary and Pancreatic Surgery, First Affiliated Hospital, School of Medicine, Zhejiang University, Hangzhou, China; 2 Department of Ophthalmology, Second Affiliated Hospital (Binjiang Branch), School of Medicine, Zhejiang University, Hangzhou, China; Kaohsiung Chang Gung Memorial Hospital, TAIWAN

## Abstract

Giant hepatic hemangioma is a benign liver condition that may be treated using surgery. We studied the digital subtraction angiographic (DSA) characteristics of giant hepatic hemangioma, and the effectiveness of transcatheter arterial embolization (TAE) alone for its treatment. This was a retrospective study of 27 patients diagnosed with giant hepatic hemangioma and treated with TAE alone (using lipiodol mixed with pingyangmycin) at the Division of Hepatobiliary and Pancreatic Surgery, First Affiliated Hospital, Zhejiang University, between January 2010 and March 2013. The feeding arteries were identified using DSA. All patients were followed up for between three weeks and 12 months. Changes in tumor diameter and symptoms were observed. The 27 patients included had giant hepatic hemangiomas ranging from 5.3 to 24.5 cm (mean, 11.24±5.08 cm) in the right (n = 13), left (n = 1) or both (n = 13) lobes. Preoperative hepatic angiography showed multiple abnormal vascular lakes in the early phase, known as the “early leaving but late returning, hanging nut on a twig” sign. On the day after TAE, hepatic transaminase levels were increased (ALT: 22.69±17.95 to 94.88±210.32 U/L; ALT: 24.00±12.37 to 99.70±211.54 U/L; both P<0.05), but not total bilirubin. Six patients complained of abdominal pain, and 12 experienced transient fever. In the months after TAE, tumor size decreased (baseline: 11.24±5.08; 3 months: 8.95±4.33; 6 months: 7.60±3.90 cm; P<0.05), and the patients’ condition improved. These results indicated that TAE was effective and safe for treating giant hepatic hemangioma. TAE may be a useful alternative to surgery for the treatment of hepatic hemangioma.

## Introduction

Hepatic hemangiomas are the most common benign tumor of the liver, accounting for 0.4–7.3% of all space-occupying hepatic lesions [1,2]. These lesions are more common in the elderly and in female patients (female:male ratio of 3:1) [1,3,4]. Hepatic hemangiomas larger than 4 cm are considered as giant hepatic hemangiomas [5,6]. Their pathogenesis is ill known, but many authors consider that they are vascular malformations or hamartomas of congenital origin that enlarge by ectasia, but not by hyperplasia or hypertrophy [7–9]. Vascular endothelial growth factor (VGEF) and IL-7 may play important roles in their formation [10–12]. The clinical features of hepatic hemangioma are: insidious onset; slow growth; asymptomatic; and favorable prognosis, with only about 5% of patients being at risk of spontaneous rupture [4,13]. Giant hepatic hemangiomas are more likely to cause symptoms than their smaller counterparts [14].

Asymptomatic lesions are usually simply observed. When symptomatic or at risk of rupture, the conventional treatment is surgery [15–19]. With the development of interventional radiology, transcatheter arterial embolization (TAE) has become a possible treatment for hepatic hemangiomas, especially for patients who are unsuitable for surgical resection. However, most of the reports published to date have used TAE to convert inoperable hemangiomas into operable ones, hence the outcomes of TAE alone are mostly unknown [13,20–24]. One study successfully used TAE alone for the treatment of cavernous hepatic hemangioma [25].

Therefore, we hypothesized that TAE can be used as the sole treatment for giant hepatic hemangiomas. The aim of the present study was to assess the characteristics and outcomes of patients diagnosed with giant hepatic hemangioma and treated with TAE only. The results of the present study could be useful for improving the management of giant hepatic hemangioma and patient outcomes.

## Materials and Methods

### Patients

This work was carried out in accordance with the Declaration of Helsinki of the World Medical Association. This study was approved ethically by the Ethics Committee of the First Affiliated Hospital, School of Medicine, Zhejiang University. Written informed consent was obtained from all patients.

This was a retrospective study of 27 patients diagnosed with giant hepatic hemangioma and treated with TAE between October 2010 and March 2012 at the Department of Surgery of the First Affiliated Hospital (Hangzhou, China). The institutional review board of the First Affiliated Hospital, Zhejiang University approved this study. Based on our clinical experience and previously published reports [14–19], the criteria used by our center to indicate treatment are a hepatic hemangioma with a maximal diameter >4 cm (irrespective of whether or not there are associated symptoms), or a smaller hepatic hemangioma that is associated with symptoms. In addition, in our center, the criterion for selection of patients for TAE alone rather than TAE plus surgery is based on a hemangioma size >5 cm. All patients underwent preoperative ultrasound, spiral computed tomography (CT) or magnetic resonance imaging (MRI). Patients were included if: 1) they had a diagnosis of giant hepatic hemangioma (>4 cm [5,6]) confirmed by ultrasound, CT or MRI; 2) digital subtraction angiography (DSA) data were available; and 3) they underwent TAE as the sole planned treatment.

### Transcatheter arterial embolization

In all patients, TAE was performed using a standard procedure by radiologists trained and experienced in this technique. The perineum was disinfected and draped with the patient in the supine position. Under local anesthesia, the right femoral artery was punctured with a 5F arterial sheath (Terumo Corporation, Japan) using the Seldinger technique, and a 5F hepatic artery catheter (RH; Cook, USA) or Yashiro catheter (Terumo Corporation, Japan) was pushed into the celiac artery, hepatic artery and superior mesenteric artery via the arterial sheath for selective angiography. After confirmation of the feeding arteries and the location, size and number of hepatic hemangiomas, a microcatheter (Terumo Corporation, Japan) was superselectively placed into the feeding arteries, and a mixture of pingyangmycin (PYM; bleomycin A5 hydrochloride for injection), iohexol contrast agent (100 ml of Omnipaque; 350 mg of iodine per ml; GM, USA) and lipiodol was slowly injected (2 ml/min) through the catheter until the periphery of the hemangioma was completely surrounded. The ratio of contrast agent to lipiodol was generally 1:3, but this was varied depending on the size of the tumor. PYM (also known as bleomycin A5) is an antitumor glycoprotein antibiotic isolated from the various components of bleomycin produced by *Streptomyces verticillus* var. *pingyangensis n*.*sp*. PYM (8–16 mg, depending on the size of the tumor) was dissolved in 2 mL of 5% glucose solution before addition to the mixed suspension of iohexol/lipiodol. Gelfoam or coils were not utilized as part of the embolization procedure.

All patients were monitored postoperatively (vital signs, oxygen saturation, routine blood investigations, liver and kidney function), with particular attention paid to the lower limb skin temperature and color, and dorsalis pedis pulses. Rehydration was considered paramount to protect the liver and prevent infection.

The patients were followed up for between three weeks and twelve months to observe for changes in tumor size, liver function and symptoms (including abdominal pain, fever and complications).

### Statistical Methods

This was an observational study and no power calculation was performed. Statistical analysis was carried out using SPSS 13.0.1 (SPSS Inc., Chicago, IL, USA). Descriptive statistics were used for continuous variables (such as hemangioma size and duration of follow-up), and are reported as means ± standard deviations (SD). Continuous variables were analyzed using analysis of variance (ANOVA). Categorical data were analyzed using Fisher exact tests. P-values <0.05 were considered statistically significant.

## Results

### Patient characteristics

Table 1 presents the clinical characteristics of the patients. Our 27 patients included 6 males and 21 females, ranging from 31 to 67 years of age (mean, 47.7±8.2 years). Hemangioma size ranged from 5.3 to 24.5 cm (mean, 11.2±5.1 cm). Hepatic hemangiomas were located in the right lobe in 13 cases (four of whom had more than one tumor), in the left lobe in one case, and in both lobes in 13 cases. In 21 cases, the hemangioma had been present for several years, but TAE was indicated because of a recent increase in tumor size (defined as a substantial enlargement of the tumor during the previous few months, with an increase in the maximal diameter of the tumor of 50% or more). In 23 of the 27 patients, the hemangiomas had been identified during medical examinations, and there were no clinical symptoms; in these patients, TAE was indicated on the basis of tumor size (>5 cm). Of the other four patients, one complained of abdominal pain and backache, one of right epigastric discomfort, fatigue and yellow urine, and one of liver pain for six months; the symptoms in these four patients disappeared after TAE. The remaining case was an incidental finding during the treatment of uterine fibroids. Among all the included patients, three had cholecystitis (two with concomitant gallbladder stones); six had an associated liver cyst; two had hypertension; one had diabetes; and one had gallbladder polyps.

**Table 1:** Characteristics of 27 patients with giant hepatic hemangioma.

Characteristic	N
Clinical presentation	
Abdominal pain	3
Recent increase in size	21
Other	3
Location of liver hemangioma	
Right liver lobe	13
Left liver lobe	1
Bilateral	13
Number of hemangioma	
1	10
≥2	17
Concomitant diseases	
Cholecystitis	3
Gallbladder stones	2
Liver cyst	6
Hypertension	2
Diabetes	1
Gallbladder polyps	1
Abdominal pain after TAE	6
Fever after TAE	12
No significant complications	9

TAE: transcatheter arterial embolization.

### Hemangioma characteristics on imaging

Hepatic hemangiomas displayed different features in the various imaging investigations. On ultrasound, hemangiomas showed as circular, oval or cloddy homogeneous hyperechoic lesions, with clear edges and acoustic shadow enhancement of the posterior wall. Some presented a small hypoechoic area in the center.

**Fig 1:**
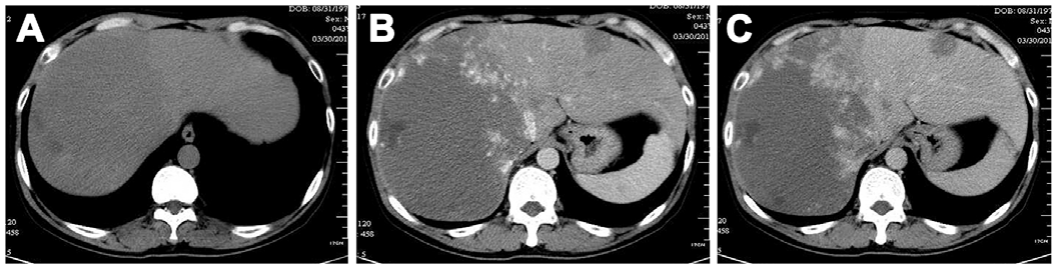
CT plain scan. An oval, low-density lesion with clear boundaries and central liquefactive necrosis (A). CT enhanced scan showing nodular enhancement at the edges of the lesion during the early arterial phase, and a higher density than the normal liver (B and C), appearing as the “fast out, slow in” sign.

CT plain scans showed round, oval or irregular low-density lesions clearly different from the liver parenchyma. CT enhanced scans showed nodular enhancement at the edges of the lesions during the early arterial phase, and higher density than the normal liver, appearing as the “early leaving but late returning sign” [26] (Fig 1).

**Fig 2:**
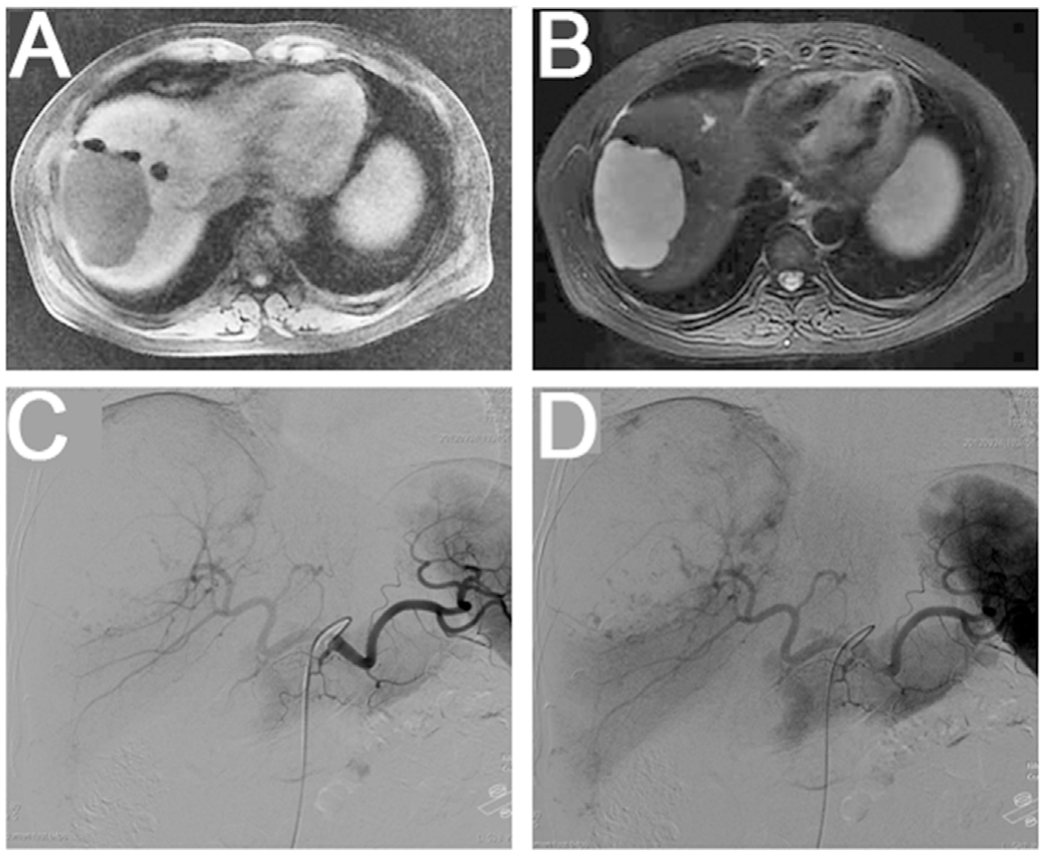
MRI scan. A giant oval mass with low signal in T1WI (A) and high signal in T2WI, appearing as the “light bulb” sign (B). DSA showing intrahepatic massive and multiple nodular tumors, developing in the early arterial phase and gradually filling to the interior, appearing as the “fast out, slow in, tree bearing fruit” sign (C and D).

MRI showed the typical liver hemangioma “light bulb sign”, i.e. high signal in T2WI against the low signal background of the liver parenchyma (Fig 2A and 2B).

Selective celiac artery, hepatic artery and superior mesenteric artery angiography showed intrahepatic slug- or popcorn-like tumor signal. In the early arterial phase, the periphery of the hepatic hemangioma was stained first, and the contrast agent gradually filled the inside of the lesion, known as the “early leaving but late returning, hanging nut on a twig” sign [26] (Fig 2C and 2D). No arteriovenous fistula or moving-portal fistula was observed.

### Transcatheter arterial embolization

**Fig 3:**
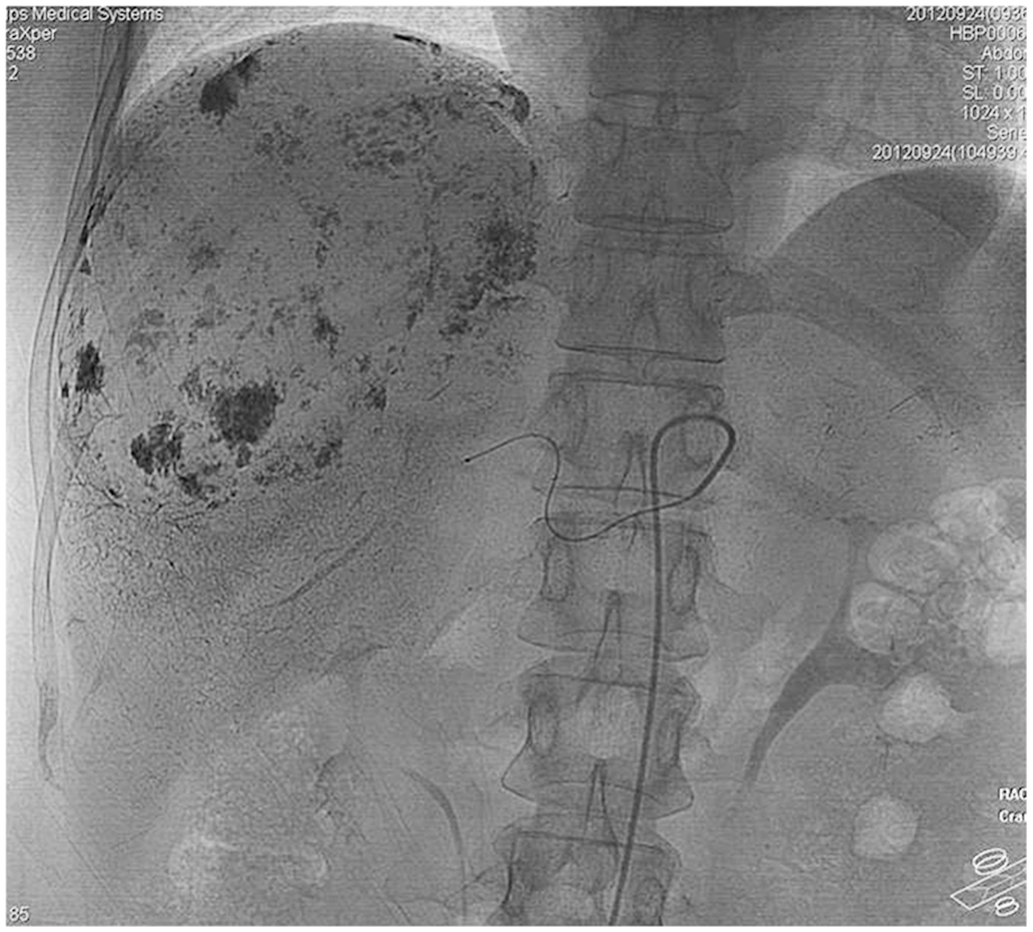
Fluoroscopy. Deposited lipiodol completely surrounding the tumor after injection of the mixture of lipiodol and PYM into the supplying artery.

**Fig 4:**
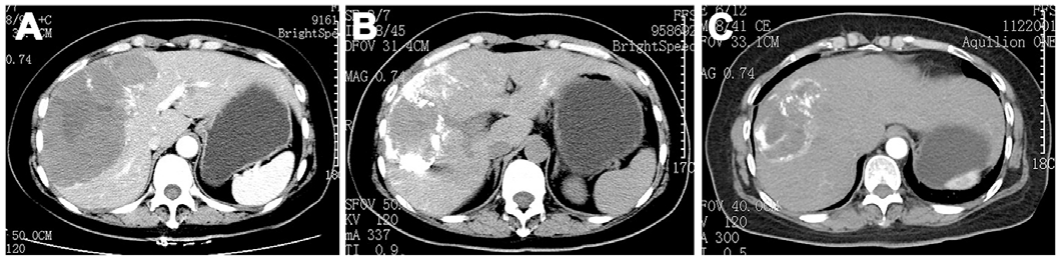
Female patient before and after TAE. (A): before TAE the hepatic hemangioma filled almost all of the right liver. Three months after TAE, the hepatic hemangioma was smaller than before TAE (B), and it became even smaller six months later (C).

All TAE procedures were considered successful, on the basis of postoperative angiography results showing that no blood reached the tumor and that lipiodol completely surrounded the tumor (Fig 3). In patients with multiple hemangiomas, each feeding artery was superselected for TAE. Twenty-one patients were injected with 8 mg of PYM, and six with 16 mg of PYM; the lipiodol volume was 2–26 ml (mean, 13.04±5.74 ml) (Fig 4).

Postoperatively, mild fever occurred in 12 patients but resolved after symptomatic treatment. An additional six patients complained of postoperative abdominal distention, and five of these also reported mild pain in the liver area. Remission of the symptoms occurred within about one week of TAE.

### Follow-up

All patients were followed up for between 3 weeks and 12 months (mean, 9.58±4.95 months). One patient was considered suitable for surgical resection of the hemangioma three weeks after TAE, due to sufficient reduction in tumor size; in this patient, there was no recurrence during the 12-months after surgery. This patient was therefore excluded from analyses of tumor size. Hemangioma size decreased significantly after TAE (P<0.05; Table 2 and Fig 4). Liver function was significantly improved the day after TAE (P<0.05; Table 3). Only one patient had a severe increase in ALT (1103 U/L) and AST (1106 U/L), but the levels decreased to 126 U/L (ALT) and 145 U/L (AST) after five days. In addition, those patients with preoperative clinical symptoms showed a postoperative alleviation of their symptoms that paralleled the decrease in tumor size. Postoperatively, hemorrhage did not occur in any of the patients.

**Table 2:** Comparison of preoperative and postoperative maximum diameters of giant hepatic hemangiomas in 26 patients.

	Preoperative	Three months postoperatively	Six months postoperatively	P
Maximal diameter (cm)	11.24±5.08	8.95±4.33	7.60±3.90	<0.05

**Table 3:** Comparison of preoperative and 1-day postoperative liver function of 26 patients with giant hepatic hemangioma.

	ALT (U/L)	AST (U/L)	TB (μmol/L)
Preoperatively	22.69±17.95	24.00±12.37	15.92±6.81
1-day postoperatively	94.88±210.32	99.70±211.54	24.38±13.54
P	<0.05	<0.05	>0.05

ALT: alanine aminotransferase; AST: aspartate aminotransferase; TB: total bilirubin.

## Discussion

The aim of the present study was to assess the characteristics and outcomes of patients diagnosed with giant hepatic hemangioma and treated with TAE only. Our 27 patients were diagnosed with giant hepatic hemangiomas ranging from 5.3 to 24.5 cm (mean, 11.24±5.08 cm). After TAE, the tumors became smaller and the patients’ condition improved.

Hepatic hemangiomas are characterized by a lack of specific clinical symptoms and insidious onset, and usually present as an incidental finding on imaging. According to the literature, ultrasound, CT and MRI detection rates for hepatic hemangioma are 57–90.5%, 73–92.2% and 97%, respectively [27–31]. DSA of hepatic hemangioma usually shows that the majority of the hemangioma is supplied by the hepatic artery, but it may also be supplied in part by other arteries, such as the phrenic artery, superior mesenteric artery and left gastric artery [32]. In addition, some hemangiomas are supplied mainly by the superior mesenteric artery [32].

It is important to differentiate hepatic hemangioma from other benign and malignant space-occupying liver lesions, including hepatic angiosarcoma, and DSA can be useful for this. First, the angiographic manifestation of hepatic angiosarcoma is usually the “early leaving but late returning” sign, and the contrast agent disappears in the lag phase. However, hepatic hemangiomas show the opposite. Second, the contrast agent penetrates rapidly into hepatic angiosarcomas, but slowly into hemangiomas. Third, the feeding arteries of hepatic angiosarcomas are often enlarged, and dysplastic vessels can be seen. However, in hemangiomas, the feeding arteries are not or only slightly enlarged, with no dysplastic vessels, but with sinusoids or vascular lakes. Fourth, huge hepatic angiosarcomas are often associated with arteriovenous fistula, moving-portal fistula and portal thrombus, while these features are rarely seen in hepatic hemangiomas. In the present study, DSA in our 27 patients with giant hepatic hemangioma showed that the primary feeding artery was the hepatic artery, without any arteriovenous fistula or moving-portal fistula.

Giant hepatic hemangiomas are those with a maximal diameter of 4 cm or more, but the necessity to treat them is still controversial [5,6]. We believe that a giant hepatic hemangioma needs treatment regardless of its location and clinical symptoms. Indeed, once a hepatic hemangioma ruptures, the mortality rate may be as high as 70% [6,33]. Furthermore, there is no consensus about the best treatment approach. The traditional view is that surgical methods such as resection and liver transplantation are superior [15–17,34]. However, with the development of interventional radiology and improvements in catheters and superselective catheterization techniques, TAE has become a very valid option. In previous reports, TAE was shown to effectively shrink the tumor, allowing for an easier resection [13,20–24]. However, it is still not established whether TAE can be used alone to treat these lesions since only one previous study has been performed, showing favorable outcomes of TAE alone for cavernous hepatic hemangioma [25]. The results of the present study strongly suggest that TAE is a feasible option in selected patients. Based on previous studies and our clinical experience, it is our opinion that TAE should be used when: 1) the hepatic hemangioma has a maximal diameter ≥4 cm; 2) the hepatic hemangioma increases in size, or is symptomatic regardless of its size; 3) the hemangioma ruptures; 4) a Kasabach-Merritt syndrome occurs; 5) the hemangioma compresses surrounding organs or intrahepatic vessels and bile duct; 6) the hemangioma is located near large vessels, hilar, subcapsular or potentially bleeding; 7) there is a high surgical risk; or 8) the patient suffers from mental stress, without clinical symptoms. Although surgery can cure hepatic hemangiomas, preoperative TAE may make surgery possible in patients with hemangioma located in the right lobe or small left lobe. In the present study, two patients with ruptured hemangioma underwent TAE, and good outcomes were achieved. One patient underwent surgical resection three weeks after TAE. Thirteen cases had hemangiomas located in both the right and left lobes, precluding surgery; these patients underwent TAE, and tumor shrinkage and symptom remission were successfully achieved.

PYM has nonspecific damaging effects on the vascular endothelium, inducing thrombi [35]. It is often used for hemangiomas in various organs [25,35–37]. Lipiodol has tumor-philic properties, and can block the tumor's blood flow [38]. Mixing PYM with lipiodol may facilitate delivery of PYM and tumor cell necrosis following thrombus formation and blocking of blood flow. In the present study, all patients were treated using this PYM/lipiodol mixture. After TAE, tumor volume significantly decreased in all patients. However, PYM can injure normal liver tissue, and so should not be used often or to repeat TAE in the short term. Twenty-five patients underwent one TAE procedure and were injected with 8–16 mg of PYM using superselective procedures into the feeding arteries of the hemangiomas; this should decrease the incidence of serious complications caused by injuries to normal liver tissue and the intrahepatic bile duct. We used doses that were utilized in previous studies [25,35–37]. Some previous investigations have used gelfoam or coils for the embolization procedure (22, 23); it will be interesting to determine in future studies whether the use of gelfoam or coils would improve the efficacy of TAE for the treatment of giant hepatic hemangioma.

The common complications of TAE for the treatment of hepatic hemangiomas are nausea, vomiting, abdominal distention, fever, hepatic dysfunction, abnormal embolization and intrahepatic bile duct injury [25,39]. In the present study, 12 patients experienced a light fever (not exceeding 39.0°C) that was relieved after symptomatic treatment. Abdominal distension and slight liver pain occurred in some patients, and may be due to embolization of tumor vessels, drug action and ischemia. ALT and AST increased after TAE, but not to a clinically significant level, except in one patient. We observed no bile duct injury or other serious complications that might be due to the superselective technique used.

The present study suffers from some limitations. First, it was a retrospective study performed in a relatively small number of patients. In addition, there was some heterogeneity in our study population. Finally, long-term follow-up was not available since most patients who were asymptomatic after TAE were unwilling to attend follow-up visits. Larger multicenter studies with longer-term follow-up should be performed to better assess TAE alone for the treatment of giant hepatic hemangiomas.

## Conclusions

In conclusion, the use of TAE for the treatment of giant hepatic hemangioma has the advantages of minimal trauma, few complications and good efficacy, especially in patients with high surgical risk. TAE therapy of giant hepatic hemangioma is safe and effective, and is an alternative to surgery.

## Supporting Information

S1 Data Raw Data. (DOCX)
