# Supplementary material for: Transcatheter Arterial Embolization Alone for Giant Hepatic Hemangioma
Source: PLoS One. 2015 Aug 19;10(8):e0135158. doi: 10.1371/journal.pone.0135158 (PMC4545419; doi:10.1371/journal.pone.0135158)
Supplement: S1 Data — (DOCX) [file pone.0135158.s001.docx]

| Characteristic | Total cases |
| --- | --- |
| Clinical presentation  abdominal pain  physical findings (recent increases)  other reasons  Location of liver hemangioma  right liver lobes  left liver lobes  bilateral  Number of hemangioma  One  two or more  Concomitant diseases  Cholecystitis  gallbladder stones  liver cyst  hypertension  diabetes  gallbladder polyps  Abdominal pain after TAE  Fever after TAE  No significant complications | 2  23（21）  2  13  1  13  10  17  3  2  6  2  1  1  6  12（all of them didn’t more than 39.0℃）  9 |

| Case  NO | Age (year) | Sex | Hospitalization | ALT  (before  TAE) | AST  (before  TAE) | TB  (before  TAE) | ALT  (Post TAE) | AST  (Post TAE) | TB  (Post TAE) | Diameter(MAX cm)  before TAE | Diameter(MAX cm) 3  month later | Diameter(MAX cm) 6  month later |
| --- | --- | --- | --- | --- | --- | --- | --- | --- | --- | --- | --- | --- |
| 1  2  3  4  5  6  7  8  9  10  11  12  13  14  15  16  17  18  19  20  21  22  23  24  25  26  27* | 31  43  52  43  48  53  54  49  31  54  66  50  48  57  49  37  46  58  57  41  53  48  45  37  46  44  51 | F  F  M  M  F  F  F  F  F  F  M  F  F  M  F  F  F  F  F  F  F  M  M  F  F  F  F | 11  17  7  18  14  22  19  14  18  11  60  14  7  6  16  6  12  35  8  7  7  5  15  10  50  9  5 | 14  11  69  20  23  12  11  10  13  9  34  18  8  68  25  14  19  13  8  12  21  44  28  13  13  60  - | 20  12  56  21  59  19  15  17  20  13  36  19  20  38  18  15  16  18  15  20  28  36  19  20  17  37  - | 11  9  17  17  15  11  18  17  22  23  40  14  8  23  9  11  25  12  7  14  14  17  17  13  14  16  - | 185  46  91  44  88  83  19  11  11  15  135  19  45  48  43  100  123  1103  8  9  62  36  25  55  15  48  - | 189  70  80  30  110  171  19  23  20  22  172  21  37  24  33  95  72  1106  20  16  74  23  19  83  33  32  - | 26  21  43  12  23  23  15  35  49  37  36  12  21  11  7  18  62  19  12  42  22  12  14  19  27  16  - | 8.4  9.5  5.8  17.6  8.7  13.8  16.7  24.5  9.0  8.2  24.0  12.4  8.0  8.2  14.5  10.1  5.3  13.5  6.0  8.6  8.5  5.4  9.4  13.6  9.0  13.6  - | 7.3  8.4  5.5  15.0  6.5  11.4  12.9  18.6  3.9  6.3  21.0  12.9  7.8  6.3  11.0  7.7  4.8  9.0  4.6  6.0  5.5  3.8  8.1  8.7  8.5  11.3  - | 6.9  7.1  4.9  13.5  5.9  9.2  12.1  15.2  2.8  5.5  18.7  11.3  6.0  5.7  8.8  6.4  3.4  7.7  3.9  5.6  3.9  2.9  6.4  5.9  8.0  10.0  - |
